# “Andropausal” Depression – biological fact or psychosocial possibility?

**DOI:** 10.1192/j.eurpsy.2023.1753

**Published:** 2023-07-19

**Authors:** C. G. Alexopoulos, J. M. Jovanovic Mirkovic, Z. Z. Jurinjak, G. Z. Golubovic

**Affiliations:** 1 Section of Cuprija, Anatomy and physiology; 2 Section of Cuprija, Biochemistry; 3 Section of Cuprija, Social and Humanities; 4 Section of Cuprija, Preclinical medicine, The Academy of Applied Preschool Teaching and Health Studies, Cuprija, Krusevac, Serbia

## Abstract

**Introduction:**

In contrast to women, men do no experience a sudden cessation of gonadal function comparable to menopause. However, there is a progressive decline in hypothalamic-pituitary-gonadal function in aging men: testosterone level decline, and there is a loss of circadian rhythm of testosterone secretion.

By age 75 years, mean plasma testosterone levels have decreased 35% compared with young adults, and more than 25% of men of this age are clinically hypogonadal. Age related hypogonadism, which has been termed»andropause«, is thought to be responsible for variety of symptoms experienced by elderly men, including reduced muscle and bone mass, sexual dysfunction, depression, fatigue and irritability.

**Objectives:**

However, it has been difficult to establish correlations between these symptoms and plasma testosterone levels. Clinical trials of testosterone replacement have documented some symptoms relief (improved muscle strength and bone mineral density), yet studies to date on the specific relation between depression and testosterone level have been methodologically flawed.

**Methods:**

Data are presented from systematic clinical and epidemiological studies with bearing on this relation:

1. population-based assessments of the relation between testosterone level, genetic factors and depression in elderly men,
2. placebo-controlled clinical trials of testosterone replacement in men with major depressive disorder.

**Results:**

Results suggest that age-related hypothalamo-pituitary-gonadal hypofunction may have particular etiologic importance in late-onset male dysthimia.

**Conclusions:**

However, there is still the dilemma whether late-onset depression in older men is predominantly biological (in which testosterone decline certainly plays an important role), psychosocial, or stress-diathesis origin.

**Disclosure of Interest:**

None Declared

